# Clinical and Radiological Analysis of Pyogenic Vertebral Osteomyelitis Immediately after Successful Antimicrobial Therapy: Considerations for Assessing Therapeutic Response

**DOI:** 10.3390/diagnostics10110861

**Published:** 2020-10-22

**Authors:** Ikchan Jeon, Eunjung Kong, Dongwoo Yu, Cheol Pyo Hong

**Affiliations:** 1 Department of Neurosurgery, Yeungnam University College of Medicine, Daegu 42415, Korea; icarus0810@hanmail.net; 2 Department of Nuclear Medicine, Yeungnam University College of Medicine, Daegu 42415, Korea; kongej@yu.ac.kr; 3 Department of Radiological Science, Catholic University of Daegu, Gyeongbuk 38430, Korea; chong@cu.ac.kr

**Keywords:** pyogenic, vertebral osteomyelitis, therapeutic response, FDG PET/MRI, SUV

## Abstract

Purpose: The clinical and radiological abnormal findings continue even after successful treatment in pyogenic vertebral osteomyelitis (PVO). We analyzed the clinical and radiological features of cured PVO based on ^18^F-fluorodeoxyglucose positron emission tomography/magnetic resonance imaging (FDG-PET/MRI) and compared the radiological differences between FDG-PET and MRI for assessing therapeutic response in PVO. Methods: This study included 43 patients (28 men and 15 women) with lumbar PVO who had no recurrence after successful antimicrobial therapy. They were divided into two groups based on the location of maximum standardized FDG uptake value (SUV_max_) of PVO lesion on FDG-PET/MRI when parenteral antibiotics were discontinued (31 in group A: Intervertebral structure; 12 in group B: Vertebral body and paravertebral muscle). The differences of clinical symptoms, hematological inflammatory indices, and radiological features were retrospectively analyzed. Results: The patients were treated with 42.28 ± 14.58 (21–89) days of parenteral antibiotics. There were significant differences in C-reactive protein (0.97 ± 1.10 vs. 0.51 ± 0.31 mg/dL, *p* = 0.041; normal range of CRP < 0.5), back pain (4.29 ± 1.13 vs. 3.50 ± 1.00, *p* = 0.040; visual analog scale), and SUV_max_ (4.34 ± 1.24 vs. 5.89 ± 1.57, *p* < 0.001) between the two groups. In the distribution pattern of PVO lesions, FDG-PET overall showed recovery pattern earlier than MRI did (*p* < 0.001). Conclusions: In cured PVO, the clinical features vary depending on the location of major structural damage of PVO lesion. The involvement of intervertebral structure is related with sustained back pain and elevation of CRP, and vertebral body/paravertebral muscle shows favorable clinical features despite advanced structural damages.

## 1. Introduction

Pyogenic vertebral osteomyelitis (PVO) is usually treated conservatively with long-term antibiotics, administered for six to 12 weeks in patients with no other complications. However, guidelines for treating PVO still remain ambiguous due to variability in treatment duration and regional antibiotic resistance [1,2,3,4]. In addition, about 50% of PVO are treated with empirical antibiotics because they are culture-negative for causative bacteria [5]. The symptoms of PVO are nonspecific, unlike those of other infections, and may not necessarily include fever [1,6]. The assessment of therapeutic response has mainly been based on clinical symptoms and hematological inflammatory indices such as C-reactive protein (CRP) and erythrocyte sedimentation rate (ESR). The clinical symptoms lack objectivity, and inflammatory indices are affected by other conditions. The clinical and radiological abnormal findings often continue even after successful antimicrobial therapy in PVO. In particular, it is hard for magnetic resonance imaging (MRI) to distinguish between residual PVO lesions and structural changes resulting from tissue damages; it can show a worsened condition compared to previous examinations because tissue damages can take several months to resolve in cases demonstrating clinical recovery [7,8]. These limitations may lead to confusion in the assessment of therapeutic response and result in the use of unnecessary antibiotics or recurrence due to insufficient treatment.

In this study, we analyzed the various clinical and radiological findings at the time of discontinuation of antibiotics and tried to find useful factors for assessing therapeutic response in the patients with lumbar PVO who had no recurrence after successful antimicrobial therapy. In particular, ^18^F-fluorodeoxyglucose positron emission tomography (FDG-PET) was used to analyze the metabolic characteristics of cured PVO lesion, and the results were compared with the clinical features and MRI findings. Recently, studies related to the use of FDG-PET to assess therapeutic response in spine infection have been reported; this approach can be less affected by the other conditions than hematological inflammatory indices [9,10,11]. Although FDG-PET has merit in identifying metabolic changes in infectious conditions, it has limited ability to visualize anatomical changes. Thus, we applied simultaneous FDG-PET/MRI for benefitting from the greater anatomical resolution of MRI plus the merits of FDG-PET while minimizing temporal and spatial errors that occur during separate applications of FDG-PET and MRI [12].

## 2. Materials and Methods

### 2.1. Patients and Data Collection

This retrospective study with clinical and radiological data in a single institution from February 2017 to February 2020 included 58 patients (36 men and 22 women). They presented with various clinical symptoms, including fever, back pain, or neurological signs with specific MRI findings of lumbar PVO as a contiguous single lesion with or without positive cultures in PVO lesion or more than two sets of blood cultures [13]. Patients were excluded if they had tuberculous spondylitis, tumors, accompanying bone infection at another site, trauma, pregnancy, concomitant severe medical problems, foreign bodies such as spinal instrumentation or bone cements of PVO lesion, or were under the age of 20 years. The patients with recurrence were also excluded. A PVO lesion comprises the upper and lower vertebrae centering on the infected disc with or without an abscess of the paraspinal muscle; this is defined as one level. If there were two infected discs with three vertebrae centering on the two infected discs, it would be defined as two levels.

All patients participated in this study with voluntary, written, informed consent, and all of the clinical and radiological data were obtained and reviewed under the approval of the institutional review board (Yeungnam University Hospital, 2016-12-019-013, and 22 December 2016). 

### 2.2. Clinical Assessment–CRP, ESR, and Visual Analog Scale (VAS)

All patients underwent clinical assessments for therapeutic responses based on the clinical symptoms and hematological indices after at least three weeks of parenteral antimicrobial therapy. The choice of parenteral antibiotics was made according to the recommendation of infectious disease physicians. VAS was used to measure back pain with 0 representing no pain to 10 representing maximum pain. Hematological inflammatory indices included CRP (normal range of < 0.5 mg/dL) and ESR (normal range of <25 mm/h).

Cure was defined as the absence of fever, improved clinical symptoms, and normalized CRP for at least six months after the discontinuation of parenteral antimicrobial therapy. Recurrence was defined as recurring back pain and/or neurological symptoms with/without fever, re-elevation of CRP, and newly developed or aggravated PVO on MRI within the follow-up period of six months.

### 2.3. Radiological Assessment–Simultaneous FDG-PET/MRI

As only patients with no recurrence were included in the study, the simultaneous FDG-PET/MRIs were thus performed immediately after successful antimicrobial therapy. Imaging data from FDG-PET/MRI were analyzed by two nuclear medicine physicians with over 10 years of experience without prior knowledge of the patients’ clinical status.

#### 2.3.1. Intensity of FDG Uptake on FDG-PET in PVO Lesion

The maximum standardized FDG uptake value (SUV_max_) was evaluated on FDG-PET/MRI performed when parenteral antimicrobial therapy was discontinued. All patients were divided into two groups based on the location of SUV_max_: Intervertebral structure (disc, endplates, and adjacent paraspinal soft tissue lesion) of groups A and vertebral body/paraspinal muscle (the bone marrow of vertebral body and paraspinal intramuscular lesion) of group B. The radiological features of the two groups are common findings of PVO lesion on the MRI in the assessment of therapeutic response. Especially, group B can be regarded as residual PVO lesion and requirement of additional antimicrobial therapy. The clinical and radiological features between the two groups were retrospectively analyzed. The locations of SUV_max_ were identified by the computerized imaging system.

#### 2.3.2. Distribution Patterns of FDG Uptake on FDG-PET, Contrast Enhancement on T1-Weighted Contrast MRI, and High Signal on T2-Weighted Fat Saturation MRI in PVO Lesion

Distribution patterns were interpreted using the criteria presented by Yu et al. [14], which were based on the distribution pattern of FDG uptake on FDG-PET, contrast enhancement on T1-weighted contrast MRI (T1C), and high signal intensity implying edema on T2-weighted fat saturation MRI (T2FS) in PVO lesion. If a PVO lesion had more than two levels, the most advanced lesion was evaluated. If there was a disagreement on the distribution pattern between two nuclear medicine physicians, the final conclusion was made after sufficient discussion. We excluded PVO patients with spinal instruments or bone cement due to the artifacts from a foreign body.

- Grade I: Activities on the bone, soft tissue, and epidural space with intensity lower than or comparable to the reference.
- Grade II: Limited activities on the margin or bulk of a destroyed disc and endplates rather than the bone, soft tissue, and epidural space with overall higher intensity than the reference.
- Grade III: Significantly increased activities on overall bone and soft tissue than the reference.

### 2.4. PET/MRI Data Acquisition

Patients fasted for at least six hours beforehand and blood glucose levels were required to be <8.9 mmol/L before the injection of FDG (3.7 MBq/kg). Integrated spinal FDG-PET/MRI (Biograph mMR; Siemens Healthcare, Erlangen, Germany) acquisition was initiated 60 min after tracer injection and patients were scanned in one–two bed positions using an approved surface coil. PET data acquisition occurred over 20 min and MRI data were simultaneously obtained using the predetermined sequence protocol.

### 2.5. Statistical Analysis

Student’s *t*-test for parametric continuous variables was used to compare two population means. Chi-squared test was used to assess the relationship between categorical variables. Statistical analyses were carried out with SPSS version 25.0 software (SPSS Inc., Chicago, IL, USA), and *p*-values <0.05 were considered statistically significant.

## 3. Results

### 3.1. Demographic Data

Among the 58 patients, 15 patients were excluded due to follow-up loss or withdrawal of participation (*n* = 2), concomitant multiple bone infections (*n* = 1), concomitant serious medical problems (*n* = 3), spinal instrumentation or bone cement (*n* = 7), and recurrence during follow-up period (*n* = 2). The final analyses were performed on 43 patients (28 males and 15 females) with a mean age of 64.58 ± 13.01 (31–85) years. The mean extent of PVO lesion was 1.21 ± 0.64 (1–4) levels, and there was no statistically significant difference between the two groups. In addition, there were also no statistically significant differences in the initial involvement of PVO lesion, vertebral body, epidural space, and paraspinal muscle. The cause of PVO was composed with 44.2% of spontaneous and 55.8% of procedure-related origins. The mean follow-up period was 11.30 ± 7.13 (6–35) months. Detailed data are described in Table 1.

### 3.2. Microorganisms and Antibiotics

The rate of microorganism identification was only 48.8% (21/43) under blood or tissue culture, and the main causal microorganism was methicillin-sensitive *Staphylococcus aureus* (MSSA). The mean duration of parenteral antibiotic therapy was 42.28 ± 14.58 (21–89) days, and there was no statistically significant difference in the duration of parenteral antibiotic therapy between the two groups (41.65 ± 14.77 vs. 43.92 ± 14.58 days, *p* = 0.652). Vancomycin was used as the final effective parental antibiotics in 44% (19/43) of the patients. It was used as a first option in nine patients who were tested culture-positive with a resistant bacterial species (Methicillin-resistant *Staphylococcus aureus* (MRSA), Methicillin-resistant *Staphylococcus epidermidis* (MRSE), and Enterococcus spp.) and in three culture-negative elderly patients who presented with unstable vital and septic condition. In the remaining cases, vancomycin was used as a second option because symptoms failed to improve or deteriorated with initial antibiotics. Details are presented in Table 2.

### 3.3. Clinical and Radiological Features of the Cured PVO Lesion

According to the location of SUV_max_, 17 cases showed SUV_max_ at adjacent paraspinal soft tissue, 14 at disc and endplates, three at vertebral body, and nine at paraspinal muscle. Finally, the patients were divided into 31 of group A and 12 of group B. In hematological indices of all patients, there were statistically significant differences in mean CRP (9.61 ± 9.32 vs. 0.85 ± 0.97 mg/dL, *p* < 0.001) and mean ESR (62.98 ± 29.50 vs. 47.98 ± 29.39 mm/h, *p* = 0.020) between the initial measures and those at the time of discontinuation of antimicrobial therapy. At the time of discontinuation of antimicrobial therapy, mean CRP of group A was statistically significantly higher than that of group B (0.97 ± 1.10 vs. 0.51 ± 0.31 mg/dL, *p* = 0.041); however, there was no statistically significant difference in mean ESR (50.61 ± 29.88 vs. 41.17 ± 28.16 mm/h, *p* = 0.351). In back pain of all patients, there was a statistically significant difference in mean VAS score between the initial measures and those at the time of discontinuation of antimicrobial therapy (7.63 ± 0.98 vs. 4.07 ± 1.14, *p* < 0.001). At the time of discontinuation of antimicrobial therapy, group A showed a statistically significantly higher mean VAS score compared to group B (4.29 ± 1.13 vs. 3.50 ± 1.00 mg/dL, *p* = 0.040). In FDG-PET, mean SUV_max_ of the PVO lesion was 4.77 ± 1.49 (2.01–8.44) in all patients, and there was statistically significant difference in mean SUV_max_ between the two groups (4.34 ± 1.24 vs. 5.89 ± 1.57, *p* = 0.001). Detailed data are described in Table 3.

### 3.4. Distribution Patterns on FDG-PET and MRI in PVO Lesion

In the distribution patterns of FDG uptake on FDG-PET, contrast enhancement on T1C, and high signal intensity on T2FS in PVO lesions of all patients, 25 of grade II and 18 of grade III in FDG-PET, eight of grade II and 35 of grade III on T1C, and 14 of grade II and 29 of grade III on T2FS were identified. There were statistically significant differences in the distribution patterns between FDG-PET and T1C (*p* = 0.013), FDG-PET and T2FS (*p* = 0.002), and T1C and T2FS (*p* = 0.001), respectively. Detailed data are described in Table 4.

## 4. Discussion

There were structural damages of intervertebral disc as the major sequela with improved paraspinal lesions such as psoas abscess after successful antimicrobial therapy in 72.1% (31/43, group A) of PVO patients. On MRI, there were sustained contrast enhancement and edematous change of intervertebral structure and adjacent soft tissues, and vertebral bodies could be observed. These indicate the formation of vascular structures and chronic inflammation presenting as the healing process after acute inflammation against infection, which tended to persist for a considerable period even after successful treatment of PVO [10,15]. However, FDG uptake on FDG-PET showed differences according to the condition of each portion within the PVO lesion in greater detail compared to MRI. In particular, the distribution pattern analysis using FDG-PET was better than MRI for earlier identification of the overall changes of PVO lesion after successful antimicrobial therapy. Higher intensity of FDG uptake was usually limited on the margin or bulk of a destroyed disc and endplates rather than the bone, paraspinal muscles, and epidural space.

We investigated pathophysiological characteristics of PVO according to the phases of osteomyelitis to understand the FDG uptake in PVO. The early phase of PVO is characterized by activated neutrophil accumulation, which uses greater amounts of glucose as its main energy source for chemotaxis and phagocytosis and is known as the respiratory burst [16]. The transport of FDG across the cellular membrane is mediated by the glucose transporter, which is more abundantly found on the cell membrane of activated inflammatory cells [17]. Consequently, FDG uptake increases as a result of activated granulocytes in the acute phase. However, in the chronic or recovery phase, lymphocytes are predominant, followed by plasma cells, histiocytes, and some polymorphonuclear leucocytes. There are formations of fibroses granulation tissues around the foci of inflammation and bone marrow, increased osteoblasts facilitating new bone formation, fatty changes, and dilated blood vessels [18]. These finding are associated with a decrease of FDG uptake from that at the early phase of PVO. Overtime, the granulation tissue disappears by apoptosis and is replaced by a mature scar within two months of injury, and FDG uptake decreases gradually as the inflammation subsides [19,20,21].

**Figure 1:**
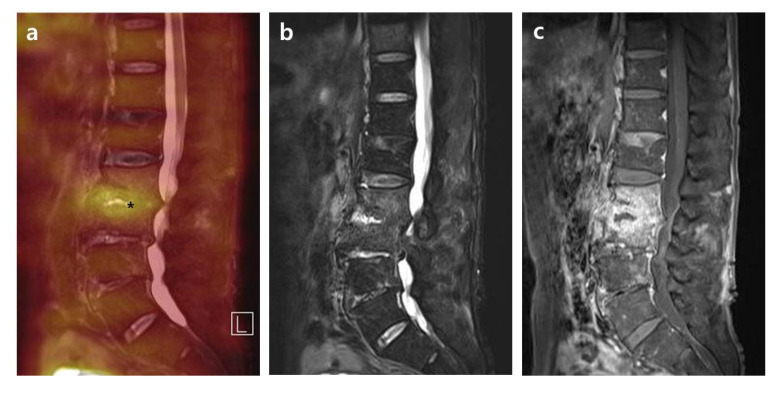
Simultaneous FDG-PET/MRI with the location of SUV_max_ on the intervertebral structure after successful antimicrobial therapy. A 78-year-old male patient shows PVO of L2–3 on simultaneous FDG-PET/MRI with SUV_max_ 3.17 on the intervertebral structure (asterisk) after 30 days of vancomycin (CRP 1.70 mg/dL). In the distribution pattern of the PVO lesion, FDG uptake on FDG-PET (**a**) and high signal intensity on T2FS (**b**) are limited on the destroyed disc and endplates rather than the bone and soft tissue. However, T1C shows significantly increased enhancements on the overall PVO lesion (**c**). FDG-PET/MRI, ^18^F-fluorodeoxyglucose positron emission tomography/magnetic resonance imaging; SUV_max_, maximum standardized ^18^F-fluorodeoxyglucose uptake value; PVO, pyogenic vertebral osteomyelitis; CRP, C-reactive protein; T2FS, T2-weighted fat saturation magnetic resonance imaging; TIC, T1-weighted contrast magnetic resonance imaging.

**Figure 2:**
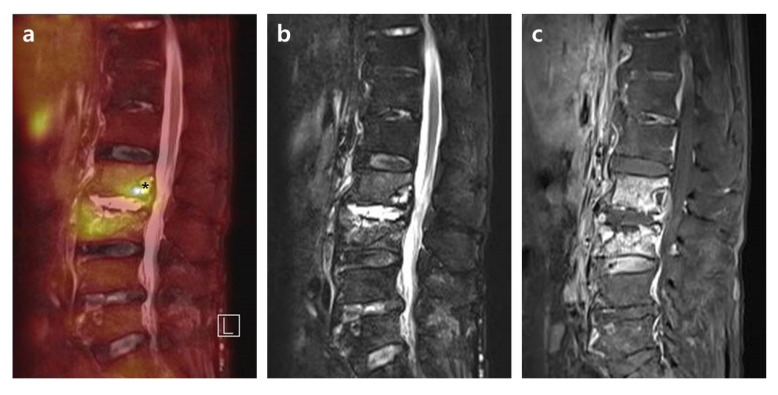
Simultaneous FDG-PET/MRI with the location of SUV_max_ on the vertebral body after successful antimicrobial therapy. A 77-year-old female patient shows PVO of L2–3 on simultaneous FDG-PET/MRI with SUV_max_ 5.99 on the vertebral body (asterisk) after 31 days of vancomycin (CRP 0.49 mg/dL). In the distribution pattern of the PVO lesion, FDG uptake on FDG-PET (**a**) shows the highest FDG uptake on the vertebral body of L2 and diffuse FDG uptake on L3. High signal intensity on T2FS (**b**) also shows similar distribution pattern of FDG-PET. However, there are significantly increased enhancements and high signal intensity on the overall PVO lesion on T1C (**c**). FDG-PET/MRI, ^18^F-fluorodeoxyglucose positron emission tomography/magnetic resonance imaging; SUV_max_, maximum standardized ^18^F-fluorodeoxyglucose uptake value; PVO, pyogenic vertebral osteomyelitis; CRP, C-reactive protein; T2FS, T2-weighted fat saturation magnetic resonance imaging; TIC, T1-weighted contrast magnetic resonance imaging.

Compared with intervertebral structures in group A (Figure 1), in group B (27.9%, 12/43) the major structural damages presenting as the abscess of vertebral body and paravertebral muscle after successful antimicrobial therapy showed more pronounced contrast enhancement and edematous change with higher SUV_max_ (Figure 2). Contrary to the FDG uptake, these lesions of group B showed an almost normalized and statistically significantly lower CRP level compared to group A. In addition, back pain also showed a similar tendency with statistical significance, as seen with CRP between the two groups. Based on the results, in PVO patients with major structural damages of the vertebral body or paravertebral muscle after successful antimicrobial therapy, therapeutic response is judged to be more accurate when it is determined by the improvement of clinical symptoms and CRP level rather than the radiological status of the PVO lesion. The fact that clinical features differ depending on the location of the major structural damage of PVO lesion after successful antimicrobial therapy can be explained by differences in the mechanical stress of each damaged portion. We expect more considerable mechanical stress to have been caused on the damaged intervertebral disc and endplates on the vertebral body or paravertebral muscle by the patient’s activities. Given these factors, the mechanical stress on the majorly damaged intervertebral structure may be related with the sustained elevation of CRP and back pain in cured PVO.

Major structural damage within the vertebral body or paravertebral muscle in advanced PVO showed pronounced contrast enhancement and edematous change with high SUV_max_ even after successful antimicrobial therapy. These findings may be related to the metabolic responses of vertebral bone marrow and paravertebral muscle in wound healing after PVO treatment. There is a report of an experimental study wherein the inflammatory stress induced by bacterial infection enhanced hematopoietic stem cell expansion with vascular proliferation and permeability in the bone marrow [22]. These specific features of bone marrow in addition to the natural healing process may be related with sustained higher FDG uptake, contrast enhancement, and edematous change. In the cases of paravertebral intramuscular abscess, increased FDG uptake continued for a long time even after successful antimicrobial therapy, which can be explained indirectly with FDG-avid abnormalities caused by surgical procedure, irradiation, and chemotherapeutics [23]. Even in sterile inflammation, FDG uptake can be taken up by neutrophils and macrophages [24,25]. In FDG-avid soft tissues in the surgical bed, it is recommended to delay FDG-PET scanning for eight weeks after surgery [26,27], which means that FDG-PET/MRIs for assessing therapeutic response in this study were conducted early in terms of FDG uptake of the healing process.

We adopted simultaneous FDG-PET/MRI for the aforementioned imaging advantages and to potentially identify specific MRI sequences linked to FDG-PET using the distribution pattern. FDG-PET showed a significantly higher frequency of grade II than MRI with predominant grade III, which means that FDG-PET has the ability to show the recovery of PVO lesion earlier than MRI. Unfortunately, we did not identify an image of grade I in both FDG-PET and MRI, which is perhaps because our FDG-PET/MRIs were performed relatively earlier (on 42.28 ± 14.58 (21–89) days of parenteral antibiotics) than in previous studies [14]. We assume that it will take more time to obtain a higher frequency of the typical pattern of FDG-PET as grades I or II after antimicrobial therapy. In the overall analysis of the distribution pattern, MRI showed a tendency of continuous extensive contrast enhancement in the cured PVO, which is not useful for earlier assessment of therapeutic response in PVO. In addition, unlike FDG-PET, MRI was unable to indicate the severity of tissue damage in the PVO lesion. We think that MRI will take considerably more time than FDG-PET to obtain a typical pattern of grade I or II after antimicrobial therapy. However, high signal on T2FS showed more frequent grade II than contrast enhancement of T1C. This means that a reduction of edematous change on PVO lesion is related with the inactivation of and decrease in activated granulocytes producing exudate during respiratory burst activation with phagocytosis in active phase under the successful antimicrobial therapy [16]. Further studies with a large number of participants are required to prove the usefulness and application of T2FS in assessing therapeutic response in PVO.

## 5. Conclusions

The clinical features of cured PVO vary depending on the location of major structural damage of PVO lesion. The involvement of intervertebral structure is related with sustained back pain and elevation of CRP, and vertebral body/paravertebral muscle shows favorable clinical features despite advanced structural damages. FDG-PET is more effective in the early assessment of therapeutic response than MRI, and the edematous change tended to be more associated with FDG-PET than contrast enhancement on MRI. We need to consider the condition of structural damage in addition to the clinical symptoms and hematological indices for assessing therapeutic response, and FDG-PET/MRI has greater merits in identifying metabolic state and obtaining anatomical resolution of PVO lesion.

## Figures and Tables

**Table 1:** Demographic and clinical data of 43 patients.

Factors	Values
**Age (years)**	64.58 ± 13.01 (31–85)
**Sex**	Male 28, Female 15
**Extent of PVO (levels)**	1.21 ± 0.64 (1–4)
Group A (*n* = 31)	1.16 ± 0.58
Group B (*n* = 12)	1.33 ± 0.78
**Involvement of PVO**	
Vertebral body	18/43 (41.9%)
Epidural space	23/43 (53.5%)
Paraspinal muscle	21/43 (48.8%)
**Cause of PVO**	
Spontaneous	19/43 (44.2%)
Procedure-related	24/43 (55.8%)
Injection or acupuncture	18/24 (75.0%)
Operation	6/24 (25.0%)
**Comorbidity**	
Diabetes mellitus	16/43 (37.2%)
Hypertension	20/43 (46.5%)
Hemodialysis	2/43 (4.7%)
Cerebrovascular disease	3/43 (6.9%)
Ischemic heart disease/arrythmia (5/2)	7/43 (16.3%)
Chronic obstructive lung disease	1/43 (2.3%)
Previous cancer history	2/43 (4.7%)
**Initial clinical symptoms**	
Fever	25/43 (58.1%)
Back pain	41/43 (95.3%)
Neurologic deficit	
Radiculopathy	24/43 (55.8%)
Weakness	7/43 (16.3%)
Bowel & bladder symptoms	1/43 (2.3%)
**Duration of follow-up (months)**	11.30 ± 7.13 (6–35)

PVO, pyogenic vertebral osteomyelitis.

**Table 2:** Characteristics of bacteriology and antibiotics.

Factors	Values
**Bacterial identification**	21/43 (48.8%)
MSSA	6
MRSA	4
Enterococcus spp.	2
MRSE	3
Streptococcus spp.	5
Klebsiella pneumonia	1
Non	22
**Bacterial diagnosis**	
Blood	6/21 (28.6%)
PVO lesion	19/21 (90.5%)
Blood & PVO lesion	4/21 (19.0%)
**Duration of parenteral antibiotics (days)**	42.28 ± 14.58 (21–89)
Group A	41.65 ± 14.78 (21–89)
Group B	43.92 ± 14.58 (24–65)
**Use of vancomycin**	19/43 (44.2%)
Culture-positive	
MRSA, MRSE, Enterococcus spp.	9
MSSA, Streptococcus spp. (as 2nd option)	2
Culture-negative	
as 1st option	3
as 2nd option	5

MSSA, methicillin-sensitive *Staphylococcus aureus*; MRSA, methicillin-resistant *Staphylococcus aureus*; MRSE, methicillin-resistant *Staphylococcus epidermidis*; PVO pyogenic vertebral osteomyelitis.

**Table 3:** Comparison of clinical and radiological features between the groups A and B.

Factors	Group A (*n* = 31)	Group B (*n* = 12)	*p* Value	Total (*n* = 43)
**Initial**				
CRP (mg/dL)	8.71 ± 8.63	11.92 ± 10.98	0.317	9.61 ± 9.32
ESR (mm/h)	61.94 ± 26.63	65.67 ± 37.12	0.715	62.98 ± 29.50
VAS score of back pain	7.48 ± 1.03	8.00 ± 0.74	0.121	7.63 ± 0.98
**When antimicrobial therapy discontinued**				
* CRP (mg/dL)	^+^ 0.97 ± 1.10	^+^ 0.51 ± 0.31	0.041	^+^ 0.85 ± 0.97
ESR (mm/h)	^+^ 50.61 ± 29.88	^+^ 41.17 ± 28.16	0.351	^+^ 47.98 ± 29.39
* VAS score of back pain	^+^ 4.29 ± 1.13	^+^ 3.50 ± 1.00	0.040	^+^ 4.07 ± 1.14
* SUV_max_	4.34 ± 1.24	5.89 ± 1.57	0.001	4.77 ± 1.49 (2.01–8.44)

PVO, pyogenic vertebral osteomyelitis; ESR, erythrocyte sedimentation rate; CRP, C-reactive protein; VAS, visual analogue scale; SUV_max_, maximum standardized ^18^F-fluorodeoxyglucose uptake value. * Statistically significant difference between two groups. ^+^ Statistically significant difference between the measures of initial and discontinuation of parenteral antimicrobial therapy; *p* values of < 0.05 were considered statistically significant.

**Table 4:** The distribution patterns of FDG uptake on FDG-PET, contrast enhancement on T1-weighted contrast magnetic resonance imaging, and high signal intensity on T2-weighted fat saturation magnetic resonance imaging.

Imagings	Groups	Grade I	Grade II	Grade III	Total
FDG uptake on FDG-PET	Group A	0	20	11	31
	Group B	0	5	7	12
	^a,b^ Total	0	25	18	43
Contrast enhancement on T1C	Group A	0	4	27	31
	Group B	0	4	8	12
	^a,c^ Total	0	8	35	43
High signal on T2FS	Group A	0	10	21	31
	Group B	0	4	8	12
	^b,c^ Total	0	14	29	43

FDG-PET, ^18^F-fluorodeoxyglucose positron emission tomography; TIC, T1-weighted contrast magnetic resonance imaging; T2FS, T2-weighted fat saturation magnetic resonance imaging; ^a^ *p* = 0.013 between FDG-PET and TIC; ^b^ *p* = 0.002 between FDG-PET and T2FS; ^c^ *p* = 0.001 between TIC and T2FS; *p* values of <0.05 were considered statistically significant.
